# Ambient Cumulative PM2.5 Exposure and the Risk of Lung Cancer Incidence and Mortality: A Retrospective Cohort Study

**DOI:** 10.3390/ijerph182312400

**Published:** 2021-11-25

**Authors:** Hung-Ling Huang, Yung-Hsin Chuang, Tzu-Hsuan Lin, Changqing Lin, Yen-Hsu Chen, Jen-Yu Hung, Ta-Chien Chan

**Affiliations:** 1 Kaohsiung Municipal Ta-Tung Hospital, Kaohsiung 801, Taiwan; 990325kmuh@gmail.com (H.-L.H.); jenyuhung@gmail.com (J.-Y.H.); 2 Division of Pulmonary and Critical Care Medicine, Kaohsiung Medical University Hospital, Kaohsiung Medical University, Kaohsiung 807, Taiwan; 3 Department of Internal Medicine, Kaohsiung Medical University Hospital, Kaohsiung Medical University, Kaohsiung 807, Taiwan; infchen@gmail.com; 4 Graduate Institute of Medicine, College of Medicine, Kaohsiung Medical University, Kaohsiung 807, Taiwan; 5 School of Medicine, College of Medicine, Kaohsiung Medical University, Kaohsiung 807, Taiwan; 6 Research Center for Humanities and Social Sciences, Academia Sinica, Taipei 115, Taiwan; yhchuang.claire@gmail.com (Y.-H.C.); sharonlin0822@gmail.com (T.-H.L.); 7 Division of Environment and Sustainability, The Hong Kong University of Science and Technology, Hong Kong, China; cqlin@ust.hk; 8 School of Medicine, Graduate Institute of Medicine, Sepsis Research Center, Center of Tropical Medicine and Infectious Diseases, Kaohsiung Medical University, Kaohsiung 807, Taiwan; 9 Department of Biological Science and Technology, College of Biological Science and Technology, National Yang Ming Chiao Tung University, Hsinchu 300, Taiwan; 10 Institute of Public Health, School of Medicine, National Yang Ming Chiao Tung University, Taipei 112, Taiwan

**Keywords:** fine particulate matter, lung cancer, health behaviors, diet

## Abstract

Smoking, sex, air pollution, lifestyle, and diet may act independently or in concert with each other to contribute to the different outcomes of lung cancer (LC). This study aims to explore their associations with the carcinogenesis of LC, which will be useful for formulating further preventive strategies. This retrospective, longitudinal follow-up cohort study was carried out by connecting to the MJ Health Database, Taiwan Cancer Registry database, and Taiwan cause of death database from 2000 to 2015. The studied subjects were persons attending the health check-ups, distributed throughout the main island of Taiwan. Cox proportional hazards regression models were used to investigate the risk factors associated with LC development and mortality after stratifying by smoking status, with a special emphasis on ambient two-year average PM_2.5_ exposure, using a satellite-based spatiotemporal model at a resolution of 1 km^2^, and on dietary habit including consumption of fruits and vegetables. After a median follow-up of 12.3 years, 736 people developed LC, and 401 people died of LC-related causes. For never smokers, the risk of developing LC (aHR: 1.32, 95%CI: 1.12–1.56) and dying from LC-related causes (aHR: 1.28, 95%CI: 1.01–1.63) rises significantly with every 10 μg/m^3^ increment of PM_2.5_ exposure, but not for ever smokers. Daily consumption of more than two servings of vegetables and fruits is associated with lowering LC risk in ever smokers (aHR: 0.68, 95%CI: 0.47–0.97), and preventing PM_2.5_ exposure is associated with lowering LC risk for never smokers.

## 1. Introduction

Lung cancer (LC) is the most common cancer in the world, with 2.2 million new cases in 2019, and it is by far the leading cause of cancer death among both men and women, making up 25% of all cancer deaths [1]. The overall incidence of LC in Taiwan ranks 15th globally and is the second highest in Asia, trailing only North Korea [1]. It has consistently been the leading cause of cancer-related death in Taiwan since 2011, with a mortality rate of 41.1 per 100,000 people in 2019 [2].

A synergic interaction of myriad risk factors contributes to the development of LC, including environmental toxin exposure, genetic predisposition, infectious comorbidities, and individual lifestyle. Despite a decline in incidence and mortality of LC following global tobacco control policies [3,4], LC among never smokers still accounts for an estimated 20% of cases in men and more than 50% of cases women [4,5]. Additionally, the global incidence of lung adenocarcinoma is still increasing against the trend of smoking rates in both sexes [4,6,7,8,9]. We can, therefore, speculate that tobacco smoke accounts for only a minority of LC development among nonsmokers. The corresponding effect of exposure to ambient fine particulate matter (PM_2.5_) on the development of LC and LC-related mortality, particularly in patients who have never smoked, has been demonstrated in several studies [10,11,12,13,14]. It is known that the impact of PM_2.5_ on LC development is greater in Asia than in Western countries [15]. In Taiwan, never smokers constitute a major proportion of LC patients, up to 50% and 90% in males and females, respectively [2,10].

A nationwide study, using a registry database, conducted in Taiwan has demonstrated a steadily increasing age-adjusted incidence of lung adenocarcinoma in male never smokers, from 9.06 to 23.25 per 100,000 population as the smoking rate decreased from 59.4 to 29.9%, and from 7.05 to 24.22 per 100,000 population in never smoker females from 1995 to 2015. The accelerated increase in PM_2.5_ levels, particularly in southern Taiwan, might be a possible explanation [10]. However, that retrospective study did not consider the impact of personal lifestyle and couldn’t reflect the temporal relationship between PM_2.5_ and LC development.

The impact of dietary factors on LC development is uncertain. A negative association between the consumption of β-carotene-, vitamin C-rich vegetables and fruits and LC development has been reported in never smoker females [16,17,18], while the opposite trend of LC increment was noted among smokers with dietary supplementation of beta-carotene [19].

With the gradual implementation of LC prevention policies around the world, this retrospective, longitudinal follow-up study has two aims: (1) to evaluate and compare the risk factors of LC incidence and LC-related mortality in ever smokers and never smokers, especially emphasizing cumulative PM_2.5_ exposure and individualized dietary habits; (2) to further investigate the factors that affect the survival of LC patients with a smoking habit vs. never smokers. Our findings provide evidence for further preventive strategy guidance.

## 2. Materials and Methods

### 2.1. Study Design and Data Source

The data for this retrospective, longitudinal follow-up cohort study were obtained from a population-based database of health examinations, the MJ Health Database (MJHD), from 2000 to 2015 in Taiwan. MJ is the name of the health check-up clinics and also the name of the database. The studied subjects were persons attending health check-ups, and they were distributed throughout the main island of Taiwan. The details of the MJHD have previously been described in the literature [20]. For each health examination visit, the results of physical examinations (anthropometric measurement and biological test data) and the results of self-administered questionnaires on lifestyle behaviors of participants were recorded. All participants signed an informed consent form before physical examination. We also linked the MJHD to Taiwan Cancer Registry (TCR) data and cause of death (COD) data for further evaluation of LC-related incidence and mortality. All participants were followed up with, from the baseline (i.e., the first health examination) until the endpoint (event of interest), the date of death, or the end of 2015. The study was approved by the Institutional Review Board (IRB) of Biomedical Science Research, Academia Sinica (IRB number: AS-IRB-BM-17044).

### 2.2. Selection of Study Population

**Figure 1:**
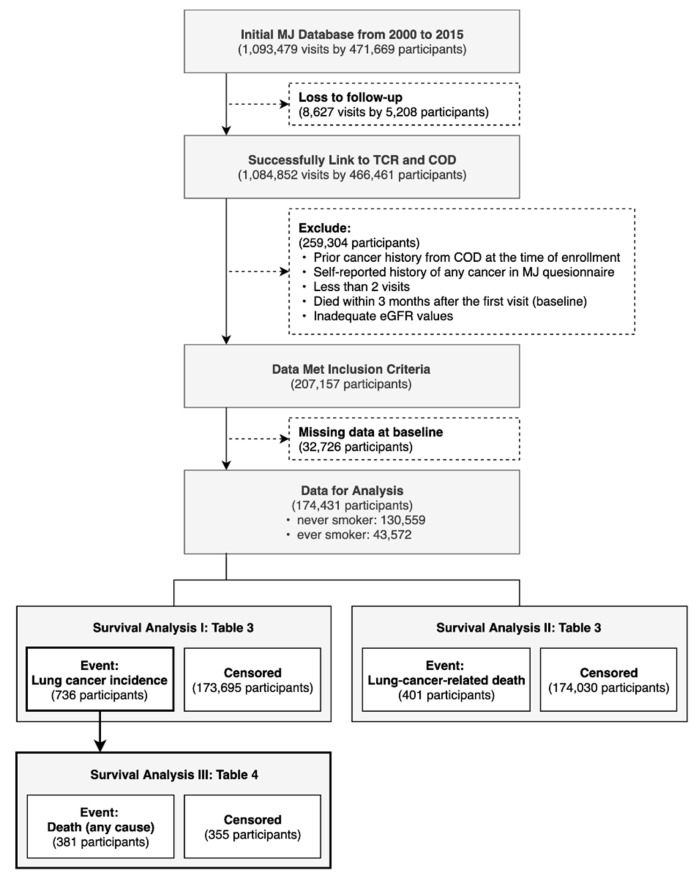
Flow Chart.

The selection process of the study population is shown in Figure 1; a total of 471,669 participants, who received at least once health examination, as shown in the MJHD, between 1 January 2000 and 31 December 2015, were enrolled. We first excluded 5208 participants with missing encrypted personal identification numbers (PIDNs), because this indicates a lack of follow-up. After linking the MJHD to the TCR and COD data through PIDNs, a total of 466,461 participants were included in the initial datasets.

Participants with cancer diagnosis or self-reported history of any cancer prior to the date of health examination or who died within three months after the first visit were excluded. We also excluded participants who had missing values on baseline covariates or inadequate estimated glomerular filtration rate (eGFR) values (i.e., eGFR < 2, eGFR≥ 200), or who provided fewer than two measuring points of eGFR during the study period, since at least two eGFR records were needed to compute an annual decline in eGFR. A total of 174,431 participants were enrolled for the final analysis, including 130,559 never smokers and 43,572 ever smokers.

### 2.3. Endpoints

For our first aim, the primary endpoint was an LC incident after the first health examination visit, which was identified from the TCR data using the International Classification of Diseases for Oncology, 3rd Edition (ICD-O-3) codes C33-C34. The secondary endpoint was LC-related death after the first health examination visit. We identified LC-related death from the COD data using the 9th revision (ICD-9) or the 10th revision (ICD-10) of the international classification of diseases (ICD-9 code 162; ICD-10 codes C33-C34).

As for our second aim, the endpoint was all-cause death of LC patients after diagnosis of LC, whose LC was diagnosed before 31 December 2014, and who were subsequently followed up until death or the end of 2015. The numbers of lung cancer cases diagnosed in different years during the studied period are listed in Table S1.

### 2.4. Definition of Variables

Information regarding participants’ demographic characteristics, lifestyle habits, family history, comorbidities, and medical history were collected via standard self-administered questionnaires and health examination records.

Diabetic mellitus was defined as fasting glucose levels >126 mg/dL or the current use of antihyperglycemic drugs. Hypertension, cardiovascular disease, and stroke were defined according to self-reported history or the current use of antihypertensive drugs and cardiac drugs. The estimated glomerular filtration rate (eGFR) was calculated from the modification of diet in renal disease (MDRD) equation [21]. The annual decline in eGFR was computed from the slope of the linear regression line of eGFR on the follow-up year. Both eGFR and CEA are common indicators in health examinations. In previous studies, eGFR decline might be associated with the incidence of some cancers, including urothelial cancer and lung cancer [22,23]. Carcinoembryonic antigen (CEA) was used as the surrogate biomarker of cancers, particularly for lung adenocarcinoma [24]. Never smokers were defined as those participants who self-reported never smoking. Participants who self-reported ever having smoked or smoking at least once a week were classified as ever smokers.

LC cases captured in our data were further classified into four types: small-cell carcinoma (ICD-O-3 morphology codes 8041, 8045), adenocarcinoma (8140, 8250, 8255, 8260, 8550, 8551, and 8560), squamous cell carcinoma (8070, 8071, and 8072), and other carcinomas. LC stages were classified into four stages (I, II, III, and IV) according to the 6th and 7th editions of the American Joint Committee on Cancer (AJCC, https://cancerstaging.org/references-tools/deskreferences/Pages/default.aspx, (accessed on 3 May 2021)).

PM_2.5_ exposure was estimated at each participant’s address reported in the questionnaire coordinates by using a satellite-based spatiotemporal model [25] with a high spatial resolution of 1 × 1 km on the basis of National Aeronautics and Space Administration (NASA) aerosol optical thickness (AOD) data [26]. The two-year mean PM_2.5_ concentration (μg/m^3^) prior to the heath examination date was used as an indicator of long-term exposure to ambient PM_2.5_ air pollution.

### 2.5. Statistical Analysis

Baseline characteristics of participants were presented as mean ± standard deviation or median (interquartile range (IQR)) for continuous variables and frequency (percentage) for categorical variables. The intergroup difference of continuous variables was compared by using the independent t-test or Mann–Whitney U test, depending on the normal distribution. A Chi-square test or Fisher’s exact test was used to compare the intergroup difference of categorical variables, as appropriate.

To investigate the effect of PM_2.5_ and fruit or vegetable consumption on the incidence and mortality of lung cancer among all study participants, Cox proportional hazards regression models were used to estimate the hazard ratios (HR) and 95% confidence intervals (95% CI) after adjusting the related covariates (details in Supplementary File S1). The cut-off points for the servings of fruits and vegetables were obtained after statistical selection since statistical significance can be only seen at 2 servings after we applied different cut-off points from 2 to 5. All statistical analyses were conducted with SAS software version 9.4 (SAS Institute, Cary, NC, USA). A *p*-value ≤ 0.05 was considered statistically significant.

## 3. Results

Overall, a total of 736 (0.42%) LC cases were identified after a median follow-up of 12.3 years. The demographic data of the enrolled population are shown in Table 1. Compared to those who did not develop LC, LC patients were more likely to be older (55.0 vs. 39.0 years old, *p* < 0.001), and had a lower education level (67.7% vs. 39.0%, *p* < 0.001), poorer renal function (77.3 vs. 86.5 mL/min/1.73 m^2^, *p* < 0.001), a higher level of carcinoembryonic antigen (CEA) (2.3 vs. 1.5 mg/dL, *p* < 0.001), greater family history of LC (8.0% vs. 5.2%, *p* < 0.001), and more comorbidities. Additionally, the LC group had more ever smokers (36.4% vs. 25.1%, *p* < 0.001) but lower PM_2.5_ exposure concentration (20.2 vs. 21.5 μg/m^3^, *p* < 0.001) than those without LC.

The characteristics of LC patients are summarized in Table 2. Compared to the LC patients who never smoked, the ever smokers were likely to be older (57.9 vs. 53.3 years old, *p* < 0.001), and male (90.3% vs. 28.9%, *p* < 0.001), and had a lower education level (77.2% vs. 62.2%, *p* < 0.001), higher CEA level (3.1 vs. 1.9 mg/dL, *p* < 0.001), less fruit and vegetable intake (87.3% vs. 96.4%, *p* < 0.001) and less long-term PM_2.5_ exposure concentration (20.0 vs. 20.3 μg/m^3^, *p* = 0.012). Most of the LC patients without smoking habits were diagnosed with adenocarcinoma (82.26%). The lag from health examination to LC diagnosis is not significantly different between the groups with different smoking habits (*p* = 0.631).

**Figure 2:**
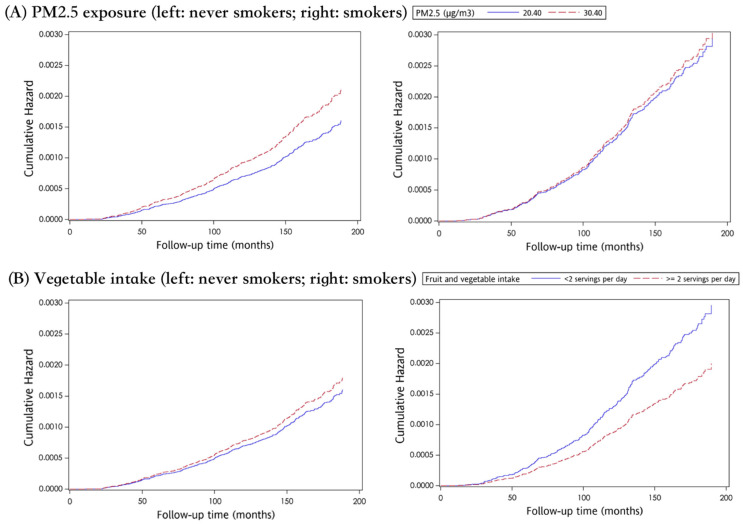
The effect of PM2.5 exposure (**A**) and vegetable intake (**B**) on cumulative hazard curve of LC incidence, stratified by smoking status.

Table 3 shows the association between each factor and both LC incidence and LC-related mortality. Ever smokers had higher cumulative incidences of LC development than never smokers (0.61% vs. 0.36%). When treating an LC incident as the event of interest, age greater than 50, lower education level, family history of LC, lower eGFR, and higher CEA level were found to be significant LC risk factors for both never smokers and ever smokers. For those who never smoked, being female was a significant risk factor for developing LC (aHR: 1.33, 95%CI: 1.07–1.64, *p* < 0.01), and a much higher proportion of adenocarcinoma was observed in never smokers than ever smokers (82.26% vs. 52.24%, *p* < 0.001, Table 2). This result is in-line with previous studies [27,28]. The effect of PM_2.5_ and vegetable and fruit intake on LC incidence differed between smokers and never smokers (Table 3 and Figure 2). For never smokers, the risk of developing LC rose significantly with every 10 μg/m^3^ increment of PM_2.5_ exposure (aHR: 1.32, 95%CI: 1.12–1.56), while such an effect was not observed among ever smokers (aHR: 0.96, 95%CI: 0.76–1.20). Conversely, consuming more than two servings of vegetables and fruits per day was able to help ever smokers reduce LC risk (aHR: 0.68, 95%CI: 0.47–0.97), yet this was not seen in never smokers.

**Figure 3:**
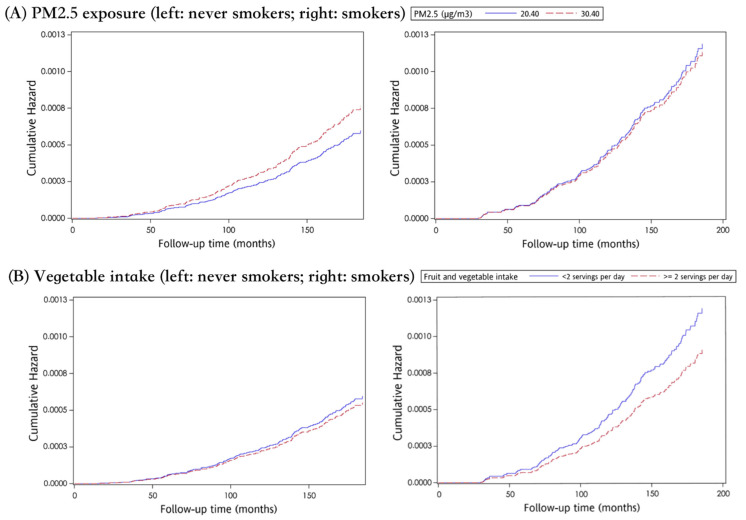
The effect of PM2.5 exposure (**A**) and vegetable intake (**B**) on cumulative hazard curve of LC-related death, stratified by smoking status.

Regarding LC-related deaths in the whole population as the event of interest, a total of 401 death events (0.23%) were reported after the median follow-up duration of 12.3 years (Table 1). Those who died of LC were older (59.1 vs. 39.0 years old, *p* < 0.001), had a lower educational level (75.3% vs. 39.0%, *p* < 0.001), were more ever smokers (46.4% vs. 25.1%, *p* < 0.001) and had more comorbidities at the baseline when compared to survivors; men accounted for around 60%, and adenocarcinoma was the commonest type (Table 1). The LC-related mortality in ever smokers was 2.63-fold higher than never smokers (0.42% vs. 0.16%), and the associations between risk factors and LC-related mortality are summarized in Table 3 and Figure 3. Every 10 μg/m^3^ increment of PM_2.5_ exposure concentration is a significant risk factor for LC-related mortality (aHR: 1.28, 95% CI: 1.01–1.63, *p* < 0.05) for never smokers, but not for ever smokers (aHR: 0.96, 95%CI: 0.76–1.20).

**Figure 4:**
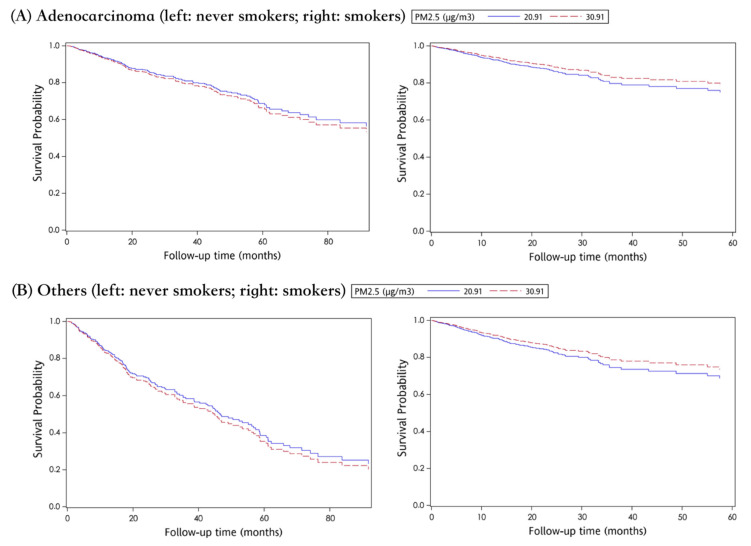
The effect of PM2.5 exposure on LC patients’ survival, stratified by smoking status and cancer type ((**A**) adenocarcinoma and (**B**) others).

Table 4 explores the association between risk factors and all-cause mortality for LC patients. Elderly age and advanced cancer stage at diagnosis of LC raised mortality risk in both never smokers (aHR: 1.03, 95%CI: 1.01–1.04, *p* < 0.001; aHR: 6.09, 95%CI: 3.87–9.57, *p* < 0.001) and ever smokers (aHR: 1.03, 95%CI: 1.01–1.05, *p* = 0.001; aHR: 7.48, 95%CI: 4.15–13.48, *p* < 0.001). For never smokers, non-adenocarcinoma type cancer (aHR: 2.55, 95%CI: 1.73–3.75, *p* < 0.001) increased mortality. For the LC patients, regardless of their smoking status, PM_2.5_ no longer seems to be a significant risk factor for death. The relationship between PM_2.5_ and mortality is plotted in Figure 4.

A total of 736 participants developed LC during the studied period (Table S2). Among them, 72 participants did not have stage information of LC from the cancer registry. The numbers of LC cases from stage 1 to stage 4 were 201 (30.27%), 30 (4.52%), 121 (18.22%), and 312 (46.99%), respectively. Higher CEA levels were noted in ever smokers who also had more comorbidities, regardless of stages of lung cancer. A higher percentage of never smokers consumed ≥2 servings of fruits and vegetables per day. The median follow-up durations from joining the database to lung cancer diagnosis were 9.5 years for the early stages of LC (stage ≤ 2), and 8.31 years for later stages of LC (stage ≥ 3), respectively.

## 4. Discussion

Several major findings were obtained from the current study. First, each 10-unit increment of cumulative PM_2.5_ exposure will increase the risk of LC incidence and LC-related mortality by 1.32-fold and 1.28-fold in never smokers, respectively, but no such increase is seen in smokers. Second, daily consumption of at least two portions of fruits and vegetables decreased by 33% the risk of LC development in ever smokers, but not in never smokers.

The current study demonstrates a visible trend of cumulative PM_2.5_ exposure which increases by 1.32 the risk of LC incidence in never smokers, consistent with the results of previous meta-analyses showing a 1–46% increase in the risk of LC incidence per 10 μg/m^3^ increase in PM_2.5_ concentration [15,29,30]. The interpretation of previous study results should be performed with caution, noting their geographical diversity and heterogeneous definitions of smoking status. Stronger associations between PM_2.5_ and LC in Asia than in North America and Europe have been reported [12,15]. The PM_2.5_ exposure concentration, with an average of 20.40 μg/m^3^, was higher in the current study than the levels reported in most previous Western studies which had average concentrations from 6.6 to 13.0 μg/m^3^ [13,29,30]; this might be a potential contributor to higher incidences of LC in Taiwan. Several possible mechanisms for the related pathogenesis of PM_2.5_ and LC development have been proposed. Under PM_2.5_ long-term exposure, the epigenetic and microenvironmental alterations, mediated by microRNA dysregulation, DNA methylation, and cell autophagy and apoptosis, may activate oncogene-associated pathways to induce carcinomatosis of the lungs [31].

In contrast, the current study shows that the association between PM_2.5_ and LC risk was insignificant in ever smokers, perhaps because cigarette smoking leads to excess body weight, which may eliminate the effect of PM_2.5_. Regarding the risk of LC development, cigarette smoking increased the relative risk by a factor of 15 to 50 in current and ever smokers, respectively, whereas the relative risks reported for PM_2.5_ seldom exceeded 1.2 [32]. One study reported that LC risk increased by 32% (95% CI: 1.02, 1.69), 20% (95% CI: 1.01, 1.41) and 16% (95% CI: 1.02, 1.30) per increase of 10 μg/m^3^ PM_2.5_ in former, current, and never smokers, respectively [15].

Previous studies have reported that each increase of 10 μg/m^3^ in the ambient concentration of PM_2.5_ is associated with a 15 to 27% increase in mortality in LC patients, particularly for former and current smokers [11,15]. However, our study shows that the impact of PM_2.5_ increment on LC-related mortality can only be seen in never smokers, rather than in ever smokers. Effects from ambient PM_2.5_ on LC incidence and mortality among ever smokers were not observed because smoking produces polycyclic aromatic hydrocarbons (PAH) contained in PM_2.5_ and PM_10_ indoors, which are directly inhaled into the body and cause health impacts [33].

Furthermore, regardless of smoking status, elderly age and advanced cancer staging at diagnosis determined the poor outcomes after LC diagnosis rather than the accumulative PM_2.5_ exposure concentration. The lack of genetic mutation reporting of LC and detailed treatment might be a potential confounder for prognosis in LC patients.

Daily consumption of at least five portions (≥400 g) of fruit and vegetables is recommended to reduce the risk of cardiovascular disease, cancer, and all-cause mortality [34,35]. The antioxidant activity contributed from biologically active compounds such as flavonoids, carotenoids, and other vitamins might be responsible for such risk reduction. However, in the literature there has not been adequate comprehensive evaluation of the protective role of fruits and vegetables in LC development and outcomes, and findings on whether they have a beneficial effect are mixed. A daily supplementation of 20–30 mg of beta-carotene has been found to increase the incidence of LC among smokers and asbestos workers [36]. A meta-analysis showed an overall of 8%−18% risk reduction for LC with daily 70–300 g fruit and vegetable intake, while the protective effect was attenuated after stratifying by smoking status, with only a marginally significant association among current smokers and an insignificant inverse trend in former or never smokers [37]. In our study, we also found a protective effect against LC in ever smokers who consumed at least two portions of fruits and vegetables daily.

There are several limitations in this study. First, the current study lacks a standardized follow-up protocol for health condition monitoring, and there are potential confounders from self-reported questionnaire data. Though environmental tobacco smoking (ETS) is an important exposure risk for LC in non-smokers, we cannot quantify the ETS effect among non-smokers due to inadequate information from questionnaires, particularly for tobacco consumption amount and duration (packs per year). Second, the exact time point of LC diagnosis is uncertain because the pre-diagnostic information of LC patients could not be obtained. Third, the two-year PM_2.5_ concentration might not reflect a direct impact on the development of lung cancer. In our case, underestimation of the risk might have occurred because PM_2.5_ was declining during the studied period [38]. For participants who later joined the health check-up program, their exposure status might be lower than earlier participants. Fourth, these datasets ignored the effects of genetic mutation of LC and also ignored the potential effects of indoor air pollution and exposure to other carcinogens on outcomes of LC. Fifth, although we have considered geographical differences in our exposure model by addressing the participants’ locations, the temporal resolution of exposure is in years. Therefore, it was hard to correlate with the community level characteristics and the variations of ambient exposure using this dataset. In southern Taiwan, the causes of serious air pollution can be attributed to three major factors including the hubs of the petrochemical industry, traffic-related air pollution, and downwind areas affecting the diffusion of air pollutants.

## 5. Conclusions

In conclusion, the strategy to lower LC incidence could differ by smoking status. For non-smokers, preventing long-term exposure to PM_2.5_ may attenuate the risk of LC development. Smoking cessation and encouraging daily consumption of at least two portions of fruits and vegetables is suggested for ever smokers.

## Figures and Tables

**Table 1:** Baseline characteristics of enrolled population, stratified by the incidence of LC and LC-related mortality.

Event of Interest	Overall (N = 174,431)	LC Incidence			LC-Related Mortality		
Variables		Non LC (N = 173,695)	LC (N = 736)	*p*-Value	Survivor (N = 174,030)	LC-Related Death (N = 401)	*p*-Value
Age (years), mean ± SD	39.02 ± 12.95	38.95 ± 12.92	54.96 ± 12.32	<0.001	38.97 ± 12.92	59.08 ± 12.05	<0.001
Female, N (%)	87,722 (50.29)	87,363 (50.30)	359 (48.78)	0.411	87,563 (50.31)	159 (39.65)	<0.001
Education level, N (%)							
High school or lower	68,233 (39.12)	67,735 (39.00)	498 (67.66)	<0.001	67,931 (39.03)	302 (75.31)	<0.001
College or higher	106,198 (60.88)	105,960(61.00)	238 (32.34)		106,099 (60.97)	99 (24.69)	
Body mass index (kg/m^2^), mean ± SD	22.91 ± 3.60	22.90 ± 3.60	23.39 ± 3.31	<0.001	22.90 ± 3.60	23.53 ± 3.40	<0.001
<18.5	16,147 (9.26)	16,111 (9.28)	36 (4.89)	<0.001	16,128 (9.27)	19 (4.74)	0.005
18.5–23.9	97,271 (55.76)	96,853 (55.76)	418 (56.79)		97,052 (55.77)	219 (54.61)	
24–27.9	46,721 (26.78)	46,501 (26.77)	220 (29.89)		(26.77)	128 (31.92)	
≥28	14,292 (8.19)	14,230 (8.19)	62 (8.42)		14,257 (8.19)	35 (8.73)	
eGFR (mL/min/1.73 m^2^), mean ± SD, N (%)	86.41 ± 17.83	86.45 ± 17.82	77.31 ± 15.91	<0.001	86.44 ± 17.82	74.44 ± 15.50	<0.001
≥ 90	64,333 (36.88)	64,187 (36.95)	146 (19.84)	<0.001	64,268 (36.93)	65 (16.21)	<0.001
60–89	105,042 (60.22)	104,530 (60.18)	512 (69.57)		104,771 (60.20)	271 (67.58)	
45–59	4471 (2.56)	4403 (2.53)	68 (9.24)		4415 (2.54)	56 (13.97)	
<45	585 (0.34)	575 (0.33)	10 (1.36)		576 (0.33)	9 (2.24)	
eGFR annual decline ≥5	20,385 (11.69)	20,310 (11.69)	75 (10.19)	0.205	20,346 (11.69)	39 (9.73)	0.221
CEA (mg/dL), mean ± SD ^a^	1.46 ± 1.18	1.45 ± 1.45	2.33 ± 3.21	<0.001	1.45 ± 1.17	2.87 ± 4.43	<0.001
Family history of LC, N (%)	9160 (5.25)	9101 (5.24)	59 (8.02)	<0.001	9136 (5.25)	24 (5.99)	0.510
Comorbidities, N (%)							
Hypertension	12,106 (6.94)	11,970 (6.89)	136 (18.48)	<0.001	12,019 (6.91)	87 (21.70)	<0.001
Diabetic mellitus	7084 (4.06)	7020 (4.04)	64 (8.70)	<0.001	7035 (4.04)	49 (12.22)	<0.001
CVD	4754 (2.73)	4696 (2.70)	58 (7.88)	<0.001	4710 (2.71)	44 (10.97)	<0.001
CVA	478 (0.27)	471 (0.27)	7 (0.95)	0.005	471 (0.27)	7 (1.75)	<0.001
Lifestyle behaviors, N (%)							
Smoking status							
Never smoker	130,559 (74.85)	130,091 (74.90)	468 (63.59)	<0.001	130,344 (74.90)	215 (53.62)	<0.001
Ever smoker	43,872 (25.15)	43,604 (25.10)	268 (36.41)		43,686 (25.10)	186 (46.39)	
Fruit/vegetable ≥ 2 servings per day	164,115 (94.09)	163,430 (94.09)	685 (93.07)	0.242	163,744 (94.09)	371 (92.52)	0.183
PM_2.5_ exposure (μg/m^3^), median ± IQR, N(%) ^b^	21.45 ± 6.95	21.45 ± 6.95	20.20 ± 6.45	<0.001	21.45 ± 6.95	20.00 ± 6.40	<0.001
<17.95	43,693 (25.05)	43,503 (25.05)	190 (25.82)		43,572 (25.04)	121 (30.17)	
17.95 ≤ PM_2.5_ < 21.45	43,671 (25.04)	43,413 (24.99)	258 (35.05)		43,532 (25.01)	139 (34.66)	
21.45 ≤ PM_2.5_ < 24.9	43,272 (24.81)	43,119 (24.82)	153 (20.79)		43,194 (24.82)	78 (19.45)	
≥24.9	43,795 (25.11)	43,660 (25.14)	135 (18.34)		43,732 (25.13)	63 (15.71)	
Follow-up time (years) ^c^, median ± IQR	12.29 ± 6.25 ^c^	12.29 ± 6.24	8.71 ± 6.00	<0.001	12.29 ± 6.25	9.43 ± 5.35	<0.001
Type of LC diagnosis, N(%) ^d^							<0.001
Small-cell	33 (0.02)		33 (4.48)		9 (0.01)	24 (5.99)	
Adenocarcinoma	525 (0.30)		525 (71.33)		305 (0.18)	220 (54.86)	
Squamous cell	76 (0.04)		76 (10.33)		26 (0.01)	50 (12.47)	
Others	102 (0.06)		102 (13.86)		39 (0.02)	63 (15.71)	

Note: Values for categorical variables are presented as number (percentage), and continuous variables are presented as mean ± standard deviation or median [1st interquartile–3rd interquartile]. *p*-Values are based on the Mann–Whitney U test for follow-up time and PM_2.5_ concentration; t-test for age, CEA level, BMI, and eGFR; or Pearson’s chi-square test for eGFR annual decline, gender, education, BMI category, eGFR category, smoking status, intake of fruits and vegetables, proportions of family history of LC, type of LC diagnosis, and comorbidities. All statistical tests were two-sided. ^a^ the normal range of CEA level is <2.5 mg/dL. ^b^ PM_2.5_ is defined as the average of PM_2.5_ exposure in the two years prior to the enrollment date. The quartile cut-off points for PM_2.5_ were PM_2.5_ < 17.95 for Q1; 17.95 ≤ PM_2.5_ < 21.45 for Q2; 21.45 ≤ PM_2.5_ < 24.9 for Q3; and PM_2.5_ ≥ 24.9 for Q4. ^c^ the follow-up time was defined as the period from the baseline to the date of LC incidence, the end of follow-up (i.e., 2015/12/31), or death prior to 2015/12/31, whichever came first. ^d^ 44 people died of lung cancer-related causes without having an LC diagnosis record. LCs were classified into four types according to the ICD-O-3 morphology codes: (1) small-cell carcinoma (codes 8041, 8045); (2) adenocarcinoma (8140, 8250, 8255, 8260, 8550, 8551, and 8560); (3) squamous cell carcinoma (8070, 8071, and 8072) and (4) other carcinomas (remaining codes). Abbreviations: BMI, body mass index; CVD, cardiovascular disease CEA, carcinoembryonic antigen; CVA, cardiovascular accident; eGFR, estimated glomerular filtration rate; LC, lung cancer; PM, particulate matter.

**Table 2:** Baseline characteristics of LC patients, stratified by smoking status.

Variable	Overall (n = 736)	Never Smoker (n = 468)	Ever Smoker (n = 268)	*p*-Value
Age (years), mean ± SD	54.96 ±12.32	53.31 ± 12.05	57.85 ± 12.28	<0.001
Female, N(%)	359 (48.78)	333 (71.15)	26 (9.70)	<0.001
Education level, N(%)				<0.001
High school or lower	498 (67.66)	291 (62.18)	207 (77.24)	
College or higher	238 (32.34)	177 (37.82)	61 (22.76)	
Body mass index (kg/m^2^), N(%)				0.621
<18.5	36 (4.89)	23 (4.91)	13 (4.85)	
18.5–23.9	418 (56.79)	274 (58.55)	144 (53.73)	
24–27.9	220 (29.89)	133 (28.42)	87 (32.46)	
≥28	62 (8.42)	38 (8.12)	24 (8.96)	
eGFR (mL/min/1.73 m^2^), N(%)				0.021
≥90	146 (19.84)	103 (22.01)	43 (16.04)	
60–89	512 (69.57)	326 (69.66)	186 (69.40)	
45–59	68 (9.24)	35 (7.48)	33 (12.31)	
<45	10 (1.36)	4 (0.85)	6 (2.24)	
eGFR annual decline ≥5 (mL/min/1.73 m^2^), N(%)	75 (10.19)	50 (10.68)	25 (9.33)	0.559
CEA ^a^ (mg/dL), mean ± SD	2.33 ± 3.21	1.89 ± 3.67	3.10 ± 1.97	<0.001
Family history of LC, N(%)	59 (8.02)	36 (7.69)	23 (8.58)	0.669
Comorbidities, N(%)				
Hypertension	136 (18.48)	83 (17.74)	53 (19.78)	0.492
Diabetic mellitus	64 (8.70)	34 (7.26)	30 (11.19)	0.069
CVD	58 (7.88)	38 (8.12)	20 (7.46)	0.750
CVA	7 (0.95)	3 (0.64)	4 (1.49)	0.252
Fruit/vegetable ≥ 2 servings per day, N(%)	685 (93.07)	451 (96.37)	234 (87.31)	<0.001
PM_2.5_ exposure (μg/m^3^), median ± IQR, N(%) ^b^	20.20 ± 6.45	20.30 ± 6.24	20.00 ± 7.77	0.012
<17.95	190 (25.82)	106 (22.65)	84 (31.34)	
17.95 ≤ PM_2.5_ < 21.45	258 (35.05)	164 (35.04)	94 (35.07)	
21.45 ≤ PM_2.5_ < 24.9	153 (20.79)	106 (22.65)	47 (17.54)	
≥24.9	135 (18.34)	92 (19.66)	43 (16.04)	
Time to event (years), median ± IQR	8.71 ± 6.00	8.55 ± 6.55	8.93 ± 5.36	0.631
Types of LC diagnosis, N(%) ^c^				<0.001
Small-cell	33 (0.02)	4 (0.85)	29 (10.82)	
Adenocarcinoma	525 (0.30)	385 (82.26)	140 (52.24)	
Squamous cell	76 (0.04)	20 (4.27)	56 (20.90)	
Others	102 (0.06)	59 (12.61)	43 (16.04)	
All-cause death, N(%)	381 (51.77)	206 (44.02)	175 (65.30)	<0.001

Note: Values for categorical variables are presented as number (percentage), and continuous variables are presented as mean ± standard deviation or median [1st interquartile–3rd interquartile]. *p*-values are based on the Mann–Whitney U test for time to event and PM_2.5_ concentration; t-test for age and CEA level; or Pearson’s chi-square test for eGFR annual decline, gender, education, BMI, eGFR category, intake of fruits and vegetables, proportions of family history of LC, type of LC diagnosis, and comorbidities. All statistical tests were two-sided. ^a^ the normal range of CEA level is <2.5 mg/dL. ^b^ PM_2.5_ is defined as the average of PM_2.5_ exposure in the two years preceding the LC diagnosis date. The quartile cut-off points for PM_2.5_ < 17.95 for Q1; 17.95 ≤ PM_2.5_ < 21.45 for Q2; 21.45 ≤ PM_2.5_ < 24.9 for Q3; and PM_2.5_ ≥ 24.9 for Q4. ^c^ LCs were classified into four types according to the ICD-O-3 morphology codes: (1) small-cell carcinoma (codes 8041, 8045); (2) adenocarcinoma (8140, 8250, 8255, 8260, 8550, 8551, and 8560); (3) squamous cell carcinoma (8070, 8071, and 8072) and (4) other carcinomas (remaining codes). Abbreviations: BMI, body-mass index; CEA, carcinoembryonic antigen; CVA, cardiovascular accident; CVD, cardiovascular disease; eGFR, estimated glomerular filtration rate; LC, lung cancer; PM, particulate matter.

**Table 3:** Independent risk factors for LC incidence and LC-related mortality, stratified by smoking status.

Event of Interest	LC Incidence				LC-Related Mortality			
	Never Smokers (N = 130,559; LC = 468)		Ever Smokers (N = 43,872; LC = 268)		Never Smokers (N = 130,559; Death = 215)		Ever Smokers (N = 43,872; Death = 186)	
Variables	aHR (95%CI)	*p*-Value	aHR (95%CI)	*p*-Value	aHR (95%CI)	*p*-Value	aHR (95%CI)	*p*-Value
Age ≥ 50	5.26 (4.16, 6.65)	<0.001	6.98 (5.09, 9.59)	<0.001	7.33 (5.05, 10.64)	<0.001	10.98 (7.09, 17.01)	<0.001
Female	1.33 (1.07, 1.64)	0.009	0.89 (0.59, 1.35)	0.577	1.14 (0.84, 1.54)	0.403	0.61 (0.34, 1.11)	0.105
Lower education level	1.05 (0.84, 1.31)	0.653	1.86 (1.37, 2.54)	<0.001	1.15 (0.82, 1.61)	0.422	2.33 (1.54, 3.53)	<0.001
Body-mass index (kg/m^2^)								
18.5–23.9	ref.		ref.		ref.		ref.	
<18.5	0.77 (0.50, 1.18)	0.232	1.22 (0.69, 2.17)	0.493	1.02 (0.54, 1.91)	0.954	1.25 (0.60, 2.58)	0.553
24–27.9	0.80 (0.64, 0.99)	0.040	0.74 (0.56, 0.97)	0.027	0.72 (0.53, 0.99)	0.042	0.81 (0.59, 1.12)	0.198
≥28	0.72 (0.51, 1.03)	0.075	0.72 (0.47, 1.12)	0.150	0.60 (0.35, 1.01)	0.054	0.77 (0.45, 1.30)	0.320
eGFR (mL/min/1.73 m^2^)								
≥90	ref.		ref.		ref.		ref.	
60–89	1.58 (1.25, 2.00)	<0.001	1.33 (0.93, 1.89)	0.117	1.88 (1.28, 2.77)	0.001	1.10 (0.71, 1.68)	0.676
45–59	1.85 (1.23, 2.79)	0.003	1.87 (1.15, 3.06)	0.012	3.13 (1.81, 5.40)	<0.001	1.92 (1.11, 3.33)	0.020
<45	1.47 (0.54, 4.06)	0.453	2.49 (1.03, 6.02)	0.043	2.37 (0.71, 7.85)	0.160	2.82 (1.13, 7.05)	0.026
eGFR annual decline ≥ 5	1.28 (0.94, 1.73)	0.117	1.27 (0.83, 1.96)	0.269	1.26 (0.79, 2.02)	0.327	1.36 (0.82, 2.27)	0.236
CEA(mg/dL), per increment	1.06 (1.04, 1.08)	<0.001	1.16 (1.11, 1.22)	<0.001	1.07 (1.05, 1.09)	<0.001	1.19 (1.13, 1.25)	<0.001
Family history of LC	1.68 (1.20, 2.37)	<0.001	1.73 (1.12, 2.65)	0.013	1.45 (0.84, 2.49)	0.184	1.07 (0.56, 2.02)	0.844
Fruit/vegetable ≥ 2 servings per day	1.12 (0.69, 1.82)	0.648	0.68 (0.47, 0.97)	0.034	0.92 (0.47, 1.80)	0.813	0.76 (0.48, 1.20)	0.241
PM_2.5_ exposure (μg/m^3^) ^a^, per 10 increment	1.32 (1.12, 1.56)	0.001	1.04 (0.86, 1.27)	0.666	1.28 (1.01, 1.63)	0.043	0.96 (0.76, 1.20)	0.714
HTN	1.17 (0.90, 1.52)	0.256	1.25 (0.90, 1.74)	0.190	1.17 (0.81, 1.68)	0.402	1.10 (0.74, 1.61)	0.638
DM	0.92 (0.64, 1.33)	0.663	0.99 (0.67, 1.47)	0.948	1.35 (0.87, 2.09)	0.176	0.99 (0.63, 1.54)	0.954
CVA	1.00 (0.32, 3.15)	0.996	1.14 (0.42, 3.12)	0.793	1.13 (0.28, 4.60)	0.867	1.72 (0.69, 4.27)	0.243
CVD	1.37 (0.96, 1.94)	0.079	1.14 (0.71, 1.83)	0.594	1.70 (1.09, 2.64)	0.019	1.42 (0.86, 2.34)	0.166

Note: The effect size of each variable was adjusted for the variables listed in Table 1 in each model. ^a^ PM_2.5_ is defined as the average of PM_2.5_ exposure in the two years prior to enrollment date. Abbreviations: aHR, adjusted hazard ratio; BMI, body-mass index; CI, confidence interval; eGFR, estimated glomerular filtration rate; LC, LC; PM, particulate matter.

**Table 4:** Independent risk factors for all-cause mortality in LC patients, stratified by smoking status.

	Never Smoker ^a^ (N = 323; Death = 161)		Ever Smoker (N = 192; Death = 131)	
Variable	aHR (95%CI)	*p*-Value	aHR (95%CI)	*p*-Value
Age at diagnosis of LC	1.03 (1.01, 1.04)	<0.001	1.03 (1.01, 1.05)	0.001
Female (ref. male)	0.67 (0.47, 0.95)	0.024	1.04 (0.57, 1.92)	0.891
Lower education level (ref. college or higher)	0.79 (0.55, 1.14)	0.210	1.03 (0.61, 1.74)	0.907
Cancer stage ^b^ III and IV (ref. I and II)	6.09 (3.87, 9.57)	<0.001	7.48 (4.15, 13.48)	<0.001
Cancer type ^c^ non-adeno(ref. adenocarcinoma)	2.55 (1.73, 3.75)	<0.001	1.29 (0.90, 1.86)	0.165
PM_2.5_ exposure ^d^, per 10 increment, μg/m^3^	1.09 (0.83, 1.44)	0.534	0.82 (0.62, 1.08)	0.154

Note: Only those whose LC was diagnosed before 31 December 2014 and who had all the covariate information were included in the statistical model. The effect size of each variable was adjusted for the other variables in each model. ^a^ Never smokers were defined as those who had never smoked. ^b^ LC stages were classified according to the American Joint Committee on Cancer (AJCC), the sixth and seventh editions. ^c^ LCs were classified into four types according to the ICD-O-3 morphology codes: (1) small-cell carcinoma (codes 8041, 8045); (2) adenocarcinoma (8140, 8250, 8255, 8260, 8550, 8551, and 8560); (3) squamous cell carcinoma (8070, 8071, and 8072) and (4) other carcinomas (remaining codes). ^d^ PM_2.5_ is defined as the average of PM_2.5_ exposure in the two years prior to LC diagnosis date. Abbreviations: aHR, adjusted hazard ratio; CI, confidence interval; LC, lung cancer; PM, particulate matter.

## Data Availability

The data that support the findings of this study are available from the MJ Health Research Foundation and Ministry of Health and Welfare, Taiwan, but restrictions apply to the availability of these data, which were under approval for the current study and so are not publicly available. The linked data set used in this study had to be analyzed in person in the Health and Welfare Data Science Center, Ministry of Health and Welfare, Taiwan.

## Supplementary Materials

The following are available online at https://www.mdpi.com/article/10.3390/ijerph182312400/s1, Table S1: The number of lung cancer cases diagnosed by year from 2000 to 2015; Table S2: The descriptive statistics for the different stages of lung cancer cases; File S1: The detailed statistical analysis and variables’ definitions.
