# Supplementary material for: Ambient Cumulative PM2.5 Exposure and the Risk of Lung Cancer Incidence and Mortality: A Retrospective Cohort Study
Source: Int J Environ Res Public Health. 2021 Nov 25;18(23):12400. doi: 10.3390/ijerph182312400 (PMC8656990; doi:10.3390/ijerph182312400)
Supplement: Supplementary file 1 [file ijerph-18-12400-s001.zip › ijerph-1446153-supplementary.pdf]

Supplementary File S1. Statistical Analysis

To investigate the effects of $PM_{2.5}$ and fruit or vegetable consumption on the incidence and mortality of lung cancer among all the study participants, Cox proportional hazard models were fitted and adjusted for the following covariates: age at the first health examination visit (≥50 or <50), sex (male or female), education level ([high school or lower] or [college or higher]), BMI (kg/m^2) (<18.5, 18.5-24, 24-28, and ≥ 28), family history of lung cancer (yes or no), vegetable and fruit intake frequency ([<2 servings/day] or ≥2 servings/day]), HTN (yes or no), DM (yes or no), CVD (yes or no), stroke (yes or no), eGFR (≥ 90, 60-90, 45-60, or <45), annual decline in eGFR (>5 or <5), $PM_{2.5}$ exposure (continuous, $\mu g/m^3$), and carcinoembryonic antigen (CEA) (continuous, mg/dL) at the first health examination visit.

To investigate the effects of $PM_{2.5}$ and fruit and vegetable consumption on mortality of lung cancer among lung cancer patients, we enrolled participants who were diagnosed with lung cancer between 1 Jan 2000 and 31 Dec 2014. All participants were followed up from the baseline until death, or the end of 2015. Cox proportional hazard models were fitted and adjusted for the following covariates: age at diagnosis of lung cancer (continuous, years), sex (male or female), education level ([high school or lower] or [college or higher]), stage at diagnosis of lung cancer ([I or II] or [III or IV]), cancer type (adenocarcinoma or others), and $PM_{2.5}$ exposures at diagnosis of lung cancer (continuous, ug/m^3).

The stratified Cox proportional hazards regression which controls by smoking status models the hazard rates for the incidence of LC and LC-related mortality, respectively, which can be specified as:

$$h_j(t \mid X_1, X_2, \ldots, X_p) = h_{0j}(t) \cdot \exp(\beta_0 + \beta_1 X_1 + \beta_2 X_2 + \ldots + \beta_p X_p), \quad j = 1, 2.$$

where j =1 or 2 stands for never smokers and ever smokers, respectively. The $h_{0j}(t)$, j= 1, 2 are unspecified baseline hazard functions: $h_{01}(t)$ for never smokers and $h_{02}(t)$ for ever smokers. Because there are different baseline hazard functions, the fitted stratified Cox model will yield different estimated survival curves of never smokers and ever smokers for the incidence of LC and LC-related mortality, respectively. The Cox regression coefficients $\beta_1, \beta_2, \ldots, \beta_p$ are estimated from the data of the incidence of LC and LC-related mortality, respectively. The covariates, $X_1, X_2, \ldots, X_p$, which are used to adjust for the hazard function, $h_j(t \mid X_1, X_2, \ldots, X_p)$, are age at the first health examination visit (≥50 or <50), sex (male or female), education level ([high school or lower] or [college or higher]), BMI (kg/m^2) (<18.5, 18.5-24, 24-28, and ≥ 28), family history of lung cancer (yes or no), vegetable and fruit intake frequency ([<2 servings/day] or [≥2 servings/day]), HTN (yes or no), DM (yes or no), CVD (yes or no), stroke (yes or no), eGFR (≥ 90, 60-90, 45-60, or <45), annual decline in eGFR (>5 or <5), $PM_{2.5}$ exposure

(continuous, μg/m^3), and carcinoembryonic antigen (CEA) (continuous, mg/dL) at the first health examination visit.

Table S1. The number of lung cancer cases diagnosed by year from 2000 to 2015

Diagnosis year	No. of lung cancer cases
2000	0
2001	0
2002	5
2003	8
2004	28
2005	23
2006	28
2007	38
2008	54
2009	64
2010	51
2011	67
2012	78
2013	97
2014	88
2015	107

Table S2. The descriptive statistics for the different stages of lung cancer cases

Variable	STAGE 1 & STAGE 2 Overall (n=231)	Never smoker (n=168)	Ever smoker (n=63)	*P* value	STAGE 3 & STAGE 4 Overall (n=433)	Never smoker (n=250)	Ever smoker (n=183)	*P* value
Age (years), mean ± SD	52.17 ± 12.23	51.07 ± 11.84	55.1 ± 12.86	0.0256	56.7 ± 12.29	55.17 ± 12.29	58.79 ± 12.01	0.0024
Female, N(%)	126 (54.55)	115 (68.45)	11 (17.46)	<0.001	198 (45.73)	186 (74.4)	12 (6.56)	<0.001
Education level, N(%)				0.0639				0.0047
High school or lower	135 (58.44)	92 (54.76)	43 (68.25)		320 (73.9)	172 (68.8)	148 (80.87)	
College or higher	96 (41.56)	76 (45.24)	20 (31.75)		113 (26.1)	78 (31.2)	35 (19.13)	
Body mass index (kg/m^2), N(%)				0.9377				0.2956
< 18.5	13 (5.63)	9 (5.36)	4 (6.35)		21 (4.85)	12 (4.8)	9 (4.92)	
18.5-23.9	138 (59.74)	101 (60.12)	37 (58.73)		240 (55.43)	148 (59.2)	92 (50.27)	
24-27.9	66 (28.57)	47 (27.98)	19 (30.19)		132 (30.48)	70 (28)	62 (33.88)	
≥ 28	14 (6.06)	11 (6.55)	3 (4.76)		40 (9.24)	20 (8)	20 (10.93)	
eGFR (ml/min/1.73m^2), N(%)				0.0084				0.3739
≥ 90	48 (20.78)	43 (25.6)	5 (7.94)		88 (20.32)	53 (21.2)	35 (19.13)	
60 – 89	162 (70.13)	114 (67.86)	48 (76.19)		291 (67.21)	170 (68)	121 (66.12)	
45 – 59	19 (8.23)	10 (6)	9 (14.29)		48 (10.88)	25 (10)	22 (12.02)	
< 45	2 (0.87)	1 (0.6)	1 (1.59)		7 (1.59)	2 (0.8)	5 (2.73)	
eGFR annual decline ≥5 (ml/min/1.73m^2), N(%)	22 (9.52)	18 (10.71)	4 (6.35)	0.3141	47 (10.85)	26 (10.4)	21 (11.48)	0.7223
CEA[a] (mg/dL), mean ± SD	1.92 ± 1.59	1.66 ± 1.56	2.59 ± 1.45	<0.001	2.61 ± 3.95	2.11 ± 4.81	3.3 ± 2.12	0.0006
Family history of LC, N (%)	21 (9.09)	14 (8.33)	7 (11.11)	0.5131	30 (6.93)	17 (6.8)	13 (7.1)	0.9021
Comorbidities, N (%)								
Hypertension	36 (15.58)	22 (13.1)	14 (22.22)	0.0885	84 (19.4)	50 (20)	34 (18.58)	0.7119
Diabetic mellitus	16 (6.93)	8 (4.76)	8 (12.7)	0.0344	41 (9.71)	22 (8.8)	19 (10.38)	0.5785
CVD	14 (6.06)	9 (5.36)	5 (7.94)	0.4643	41 (9.47)	27 (10.8)	14 (7.65)	0.2688
CVA	2 (0.87)	1 (0.6)	1 (1.59)	0.4686	4 (0.92)	1 (0.4)	3 (1.63)	0.183
Fruit/vegetable ≥ 2 servings per day, N(%)	210 (90.91)	159 (94.64)	51 (80.95)	0.0013	405 (93.53)	242 (96.8)	163 (89.07)	0.0012
$PM_{2.5}$ exposure (μg/m^3), median ± IQR, N(%)[b]	20.2 ± 6.65	20.15 ± 6.4	20.2 ± 6.75	0.9235	20.2 ± 6.9	20.3 ± 6.15	19.9 ± 9.2	0.0066
< 17.6	59 (25.54)	42 (25)	17 (26.98)		110 (25.4)	51 (20.4)	59 (32.24)	
17.6 ≤ $PM_{2.5}$ < 22.95	103 (44.59)	76 (45.24)	27 (42.86)		193 (44.57)	116 (46.4)	77 (42.08)	
22.95 ≤ $PM_{2.5}$ < 25.2	32 (13.85)	24 (14.29)	8 (12.7)		58 (13.39)	35 (14)	23 (12.57)	
≥25.2	37 (16.02)	26 (15.48)	11 (17.46)		72 (16.63)	48 (19.2)	24 (13.11)	
Time to event (years), median ± IQR	9.5 ± 5.54	9.35 ± 5.89	9.7 ± 4.26	0.9191	8.31 ± 5.81	8.3 ± 6.44	8.55 ± 5.5	0.5487
Types of LC diagnosis, N(%)[c]				<0.001				<0.001
Small-cell	2 (0.87)	0 (0)	2 (3.17)		27 (6.24)	2 (0.8)	25 (13.66)	
Adenocarcinoma	192 (83.12)	148 (88.1)	44 (69.84)		290 (66.97)	205 (82)	85 (46.45)	
Squamous cell	19 (8.23)	5 (2.98)	14 (22.22)		52 (12.01)	12 (4.8)	40 (21.86)	

Others	18 (7.79)	15 (8.93)	3 (4.76)		64 (14.78)	31 (12.4)	33 (18.03)	
All-cause death, N(%)	40 (17.32)	25 (14.88)	15 (23.81)	0.1102	314 (72.52)	167 (66.8)	147 (80.33)	0.0018

Note: There were a total of 736 participants developing lung cancer during the studied period. Among them, 72 participants did not have stage information of lung cancer from the cancer registry. The number of lung cancer cases from stage 1 to stage 4 are 201 (30.27%), 30 (4.52%), 121 (18.22%) and 312 (46.99%), respectively.
